# Toxicological Analysis of Insects on the Corpse: A Valuable Source of Information in Forensic Investigations

**Published:** 2018-09-30

**Authors:** Mojtaba Salimi, Yavar Rassi, Omid Chatrabgoun, Artin Kamali, Mohammad Ali Oshaghi, Vida Shiri-Ghaleh, Mehrdad Moradi, Sayena Rafizadeh, Kameran Akbarzadeh, Seyedeh Zahra Parkhideh

**Affiliations:** 1 Department of Medical Entomology and Vector Control, School of Public Health, Tehran University of Medical Sciences, Tehran, Iran; 2 Department of Statistics, Faculty of Mathematical Science and Statistics, Malayer University, Malayer, Iran; 3 Department of Forensic Toxicology, Legal Medicine Center of Kermanshah Province, Kermanshah, Iran; 4 National Institute for Medical Research Development (NIMAD), Tehran, Iran

**Keywords:** Morphine, Postmortem, Necrophagous, Rabbit, Larvae

## Abstract

**Background:**

Entomotoxicology as a subset of forensic entomology can be used by analysis of carcass feeding insects to detecting of drugs or toxins, as well as the cause and manner of death in cases of ante-mortem drugs intoxication. Morphine is one of the deacetylate metabolites of heroin. The aim of this study was to determine the presence and quantity of morphine in insects on the carcass and compare them with decomposing carcass.

**Methods:**

Field of this study was in Chalabeh District and toxicological tests were carried out at the Department of Forensic Toxicology, Legal Medicine Center, Kermanshah, Iran in 2017. Morphine was inoculated into live rabbit as experimental model at concentrations of 12.5, 25, 50mg/ml, similar to those normally encountered in human overdoses, then quality and quantity of morphine were determined in insects such as *Chrysomya albiceps* (as the first wave of insect succession on human cadavers) fed on carcass.

**Results:**

Quantitative assessment at larvae showed that morphine was detected in all larvae (feeding and post feeding stage) fed on tissues from carcasses administered morphine, except for post-feeding larvae from R1 which received 12.5mg/ml dosage of morphine.

**Conclusion:**

Necrophagous insects are an indicator on the scene of crime and a potential source of information about the antemortem situation. Detection of drug in insects which is actually a reflection of the cause of death is possible.

## Introduction

In forensic entomology using the insects on the carcass, we can find various factors such as time of death, climatic condition, death location and presence of drugs or toxins in the body (1, 2). The presence of drugs in the carcass can effect on the growth and morphology of insects on the corpus with the result that some error can occur in the estimation of postmortem interval (3–9). There is a correlation between drug concentration in substrate and bred insects’ development on the substrate.

However, the pharmacokinetic of drug in insects depends on species, development stage, mode of action of drug, absorption, redistribution, and metabolism of drug and drug stability (1, 4, 10–16). Morphine is one of the heroin metabolites, and the pharmacokinetics of the opiate groups is similar to each other. We have chosen morphine as our initial drug study in this research, as heroin addiction is a major public health problem worldwide; moreover, deaths through heroin overdose are frequent. In this study morphine and its metabolites in rabbit carcass, *Chrysomya albiceps* (as the first and most abundant insects on the body) (Diptera: Calliphoridae) and *C. maxillosus* (Celoptera: Staphylinidae) were analyzed. In order to analysis of drug or toxin, different methods and devices including Radioimmunoassay (RIA), high performance liquid chromatography (HPLC), Liquid chromatography–mass spectrometry (LC-MS), enzyme immunoassay (EIA), Gas chromatography-mass spectrometry (GC-MS), thin-layer chromatography (TLC), Liquid-Chromatographie Mass enspektometrie/Mass enspektometrie (LC-MS/MS) and liquid chromatography combined with positive electrospray ionization tandem mass spectrometry (LC/ESI+MS/MS) were used due to accuracy, sensitivity and economy (7, 10, 15, 17–21).

The aim of this study was to determine the presence and quantity of morphine in insects on the carcass and compare them with decomposing carcass. Moreover, the relationship between quantity of morphine in carcass tissue, immature of *C. albiseps* (first level of food chain) and adult of *Creophilus maxilosus* (second level of food chain) was investigated.

## Materials and Methods

### Site and period of study

This study was conducted at Bisetoon (challabeh), located 13km from Kermanshah Province, west of Iran between N 34° 24′ 37″ northern latitude and E 47° 14′ 42″ eastern longitude, in 2017.

### Morphine

Morphine is the main opiate alkaloid, and the derivation of the opioid group belongs to the strong group of agonists. The melting point of morphine is 254–256 °C. A total of 90% of morphine is metabolized in the liver, and the human body excretes it through the urine. This type of drug aggregates in most of tissues. Virtually all opioid drugs are metabolized by the cytochrome P450 liver enzymes. Before being excreted by kidneys they change into glucuronide conjuges (M3G, M6G). A small amount of the drug is also excreted by the kidneys with no change. The protein binding is very low, and the half-life of the drug equals 2–3h. Morphine exerts its effects via δ, κ, μ receptors.

### Animal and Morphine Dosing

Accordance with the ethical standards of Tehran University of Medical Sciences Ethics Committee, (with Code of ethics IR.TUMS. SPH.REC.1395.1634) for toxicological analysis, three rabbits with a similar weight (R1= 2500g, R2= 2700g, R3= 3000g) as the treatment group and the fourth rabbit (R0= 2600 gr) as the control group were selected. Treatment groups were administered concentration of R1= 12.5, R2= 25 and R3= 50mg/ml of morphine sulfate (10.1, 20.2 and 40.4mg of free morphine equivalent, respectively). Morphine sulfate was diluted in 150mL isotonic saline solution and for the control group, only 150ml of isotonic saline solution (without morphine) were inoculated for three hours through the superficial vein of the ear.

**Fig. 1.**
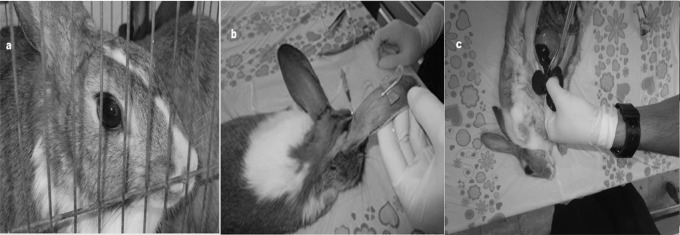
Rabbit model and Perfusion of Morphine sulfate (12.5, 25 and 50mg/ml) via the main artery of the ear (a, b). Autopsy of the treatment groups (c)

Morphine perfusion was used with scalp vein set (with specification, item No. HTF 0324S, Gauge 25G, OD 0.5mm, Color Code Orange). The perfusion bottle was placed 1.75m above the rabbit. The effect of morphine inoculation of rabbits as hypothermia, miosis and respiratory depression were observed. In order to maximum the distribution of the drug after 30–60min the rabbits were killed using chloroform. These dosages, samplings of rabbit tissue, the interval between the completion of inoculation and the death of rabbit’s, duration and rate of inoculation of morphine were calculated from pharmacokinetics (10, 22–24). The rabbit's internal tissues were sampled to determine the presence of morphine and this autopsy was performed 24h after death to create the reality conditions (Fig. 1).

Morphine was measured from internal tissues such as liver, spleen, mesenteric muscle, bile marrow at the rate of 10–20gr being kept into tubes containing 150ml deionized distilled water at the degree of 4 °C until the analysis time. The carcasses were stitched up after the autopsy and immediately transferred to the study field.

### Preparation of the specimen for TLC and HPLC analysis

*Chrysomya albiceps* and *C. maxillosus* as the dominant species on the rabbit carcass were used to analyze the toxicology. Each larval sample comprised of 30 larvae and was divided into peak feeding and post feeding. Moreover, each beetle sample contained 10 adult beetles considered for each concentration. To avoid the alteration of morphine concentration by drug metabolism in larval body, the larvae were immediately killed using hot water. The samples were kept in tubes containing deionized distilled water at the degree of 4 °C, and it was transferred to the forensic toxicology laboratory of Kermanshah located in the western part of Iran for the purpose of toxicology analysis.

### Morphine extraction of tissue, larvae, and beetle

At first, the samples were homogenized in the laboratory. Some 100ml of acidic ammonium sulfate solution was added to some 50ml of the homogenized samples (250g of ammonium sulfate in one liter of 20% hydrochloric acid). It was shaken for 20min and then exposed to heat in oven at the degree of 80 °C for 2h. By using ammoniac, the pH of the filtered solution reached 8.9. Then 100ml chloroform- isopropanol at the rate of (80:20v/v) was added to the samples, and they were shaken for 20min. Afterward, it was poured into decanter and underneath organic phase which contained morphine separated at beaker. After drying, 1ml methanol was added to the sample, and by Syringe 0.2 Micron the sample was filtered and transferred to 1.5ml polypropylene Eppendorf tube then 20μl of it was injected into the Chromatographic system.

### Thin-layer Chromatography data for morphine

TLC contained a stationary phase including absorbent material silica gel, 20×20cm in diameter, and 0.20mm thickness on the fixed base was made of glass. The standard used in the study contained 25 microliter opium, and the samples of each concentration were spotted separately at the rate of 10 microliters at one edge of a plate. Then TLC plates were put into the TLC tank which was a closed container containing a mobile phase (a combination of organic solvents), and the samples were separated at the mobile phase. The Chromatography tank was made of glass in 21.5 by 9.5 by 22cm of diameters and contained ethyl acetate 85ml, ammonia 5ml in final volume of 100ml. We kept waiting for 10min until the solvent in the tank was saturated, and we put spotted TLC plates in the tank, and the tank lid kept closed until the end of the experiment. After the solvent ascended to the top of the plate, we took it out of the tanks to be dried completely. TLC plates through Iodoplatinate acid were sprayed, and developed spots were compared with morphine standard spots. The quantities of microgram to picogram can be separated by TLC and characterized by their Rf^1^ value. Actually, Rf is equal to the distance traveled by morphine divided to the distance traveled by the solvent. In this study, opium was used as a standard for detecting morphine spots.

### Standard Solutions

Standard curves (calibration standard) were diluted by using the main sample of morphine sulfate with a 10mg/ml concentration and a 1ml volume. The dilutions of 1, 5, 10, 50 and 100ng/ml were provided from the main sample. By, injecting each dilution into the device, the area under the curve and morphine retention time were obtained.

### HPLC Data

In the course of passing through the filter in the shuffled vials, the samples collected in order to be inject into the HPLC device with the following features: KNAUER model (made in Germany), Dimension: 250 by 4.6mm with pre-column, Batch NO: B195975, Column SN: WH62, Packing Eurospher 100-5 C18, PDA detector. The mobile phase included phosphate buffer and acetonitrile with a ratio of 3:97, pH= 2.3 and the flow rate of 0.8ml/min. The injection volume of 20 microliters for each analysis turn was carried out using 100 microliter Hamilton syringe. Total run time analysis was considered to be 30min. Then all data obtained were collected for the purpose of statistical analysis.

## Results

### Thin-Layer Chromatography Results

**Fig. 2.**
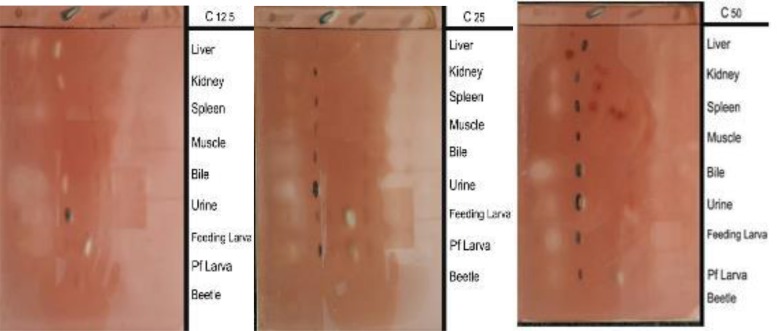
Thin-layer Chromatography of Morphine in tissues, larvae, and beetle in different dosages (12.5, 25 and 50mg/ml)

We used the TLC method for the qualitative assessment of the morphine and its presence in treated tissues with different concentrations. The observed results associated with this method in different concentrations were indicative of the morphine in rabbit tissues and *C. albiceps* larval in treated samples. In R3 groups, liver, kidney, spleen, muscle, bile, urine tissues, peak feeding and post-feeding larvae were all positive and adult *C. maxillosus* was revealed to be negative. In the concentration related to R2= 25mg/ml, liver, kidney, spleen, bile, urine, peak feeding larvae were shown to be positive, but muscle, post-feeding larvae and *C. maxillosus* beetles turned out to be negative. As for the concentration associated with R1= 12.5mg/h except for the urine sample other samples were negative (Fig. 2).

### Method Validation

In order to verify the data obtained from the morphine analysis, the efficiency of the extraction method and accuracy of the device readings, first we assessed a specific set of parameters in order to ensure reliable results were obtained.

#### Selectivity:

**Fig. 3.**
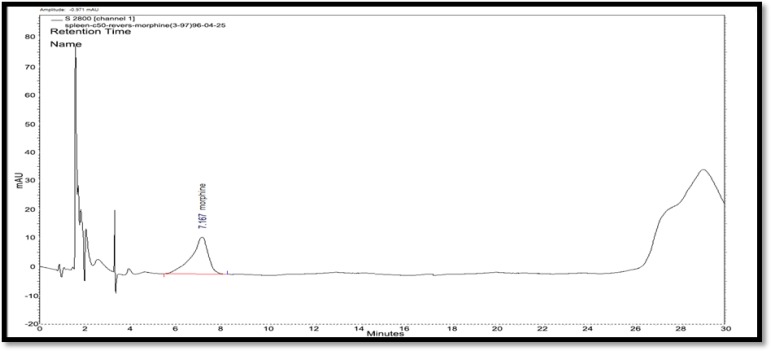
Chromatograms HPLC at Retention time 7.167min for morphine sample

**Fig. 4.**
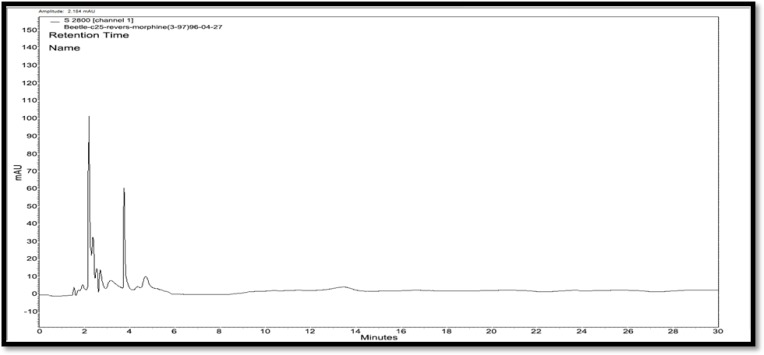
Placebo sample that indicates no interfering peaks at the retention time of morphine

The ability of the method to provide straight and clear assessment of morphine versus other component substances was determined by the selectivity parameter. The specificity of the method was examined by comparing the chromatogram of the standard and control sample. There were no interfering peaks in the control sample at the retention time of morphine (Fig. 3, 4). Retention time of morphine was approximately 7.167min. The total run time for each sample injection was about 30min.

#### Linearity:

**Fig. 5.**
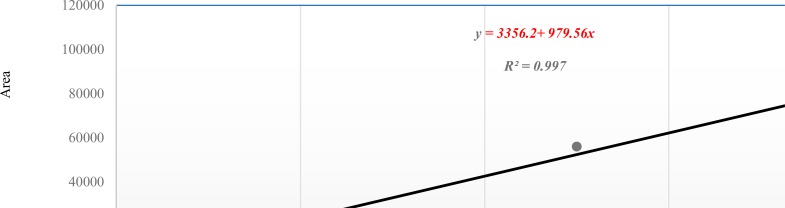
Calibration graph and Morphine sulfate line equation

This parameter was used to show the relationship between concentration and resultant signal. For this purpose, six concentrations including 1, 5, 10, 50, 100ng/ml from the standard sample were provided, and the area under the curve of each concentration with three replications was analyzed. Calibration chart and linear equation of morphine concentrations in three consecutive injections equal Y= 3356.2+979.56X where y equals the peak area of the morphine and x is indicative of morphine concentration. The correlation coefficient was R^2^ > 0.99 (Fig. 5).

#### Recovery:

Three concentrations 5, 50 and 500ng/ml were added for on three occasions to the larvae matrix in the control group which was lacking morphine, and after the extraction process the concentrations were analyzed at each concentration level. Calibration curves for the plasma assay developed with peak area of morphine (y) versus drug concentration (x) were found to be linear in the examined concentration range. The values obtained for the % recovery concentrations ranged from 98–102%.

Limit of detection and quantitation: the minimum of the observable value in the device which must, in fact, be a peak equivalent to the three-time device noise was calculated by this formula:

LOD= 3.3* (SD/S). The minimum morphine content in the sample determined with acceptable accuracy and precision was calculated by this formula LOQ= 10*(SD/S). Where *S* is the slope of the calibration curve and SD is standard deviation of the response (Table 1).

**Table 1.** Range of detection and quantitation of morphine by HPLC-UV

**Analysts**	LOD^*^ (ng/ml)	LOQ^**^ (ng/ml)
**Morphine**	8.36	25.3

* Limit of detection

** Limit of quantitation

Precision and Accuracy: the precision in the analysis method was measured through injecting three dilutions (30, 50, 100ng/ml) of morphine in three replications (n= 3) on the same day (intra-day) and three concentrations in three separate days, each day with three replications for each dilution (inter-day). Such parameters as mean concentration (C), Accuracy (Bias), standard deviation of the mean concentration of morphine (SD), coefficient of variation value [CV%= (SD/C) *100%] are shown by Table 2. The rate of CV% < 3 was calculated for both inter and intra-day parameters.

**Table 2.** The intra- and inter-day precision which proves the acceptable accuracy and precision of the method developed

**Concentration added (ng/ml)**	**Concentration founded (ng/ml)**	SD^*^	CV%^**^	**Bias%**	**Recovery**	n^***^
**Intra-day**						
30	28.3	2.00	7.14	4.3	93.3	3
50	51.1	1.13	2.23	2.2	102	3
100	101	2.35	2.37	1	101	3
**Inter-day**						
30	28.3	2.00	7.52	4.3	92	3
50	50.51	1.13	2.23	1.02	101	3
100	99.4	2.35	2.37	−0.6	99.4	3

* Standard deviation of the response

** Coefficient of variation value

*** repeat number of test

### HPLC Results of Rabbit Samples and Insects

**Fig. 6.**
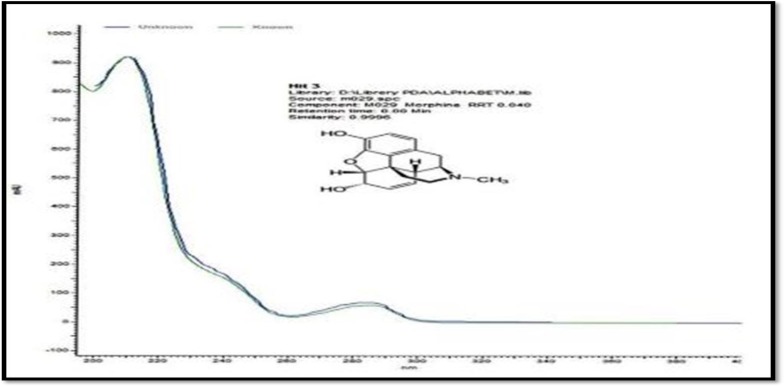
Ultraviolet Spectrum of morphine

**Fig. 7.**
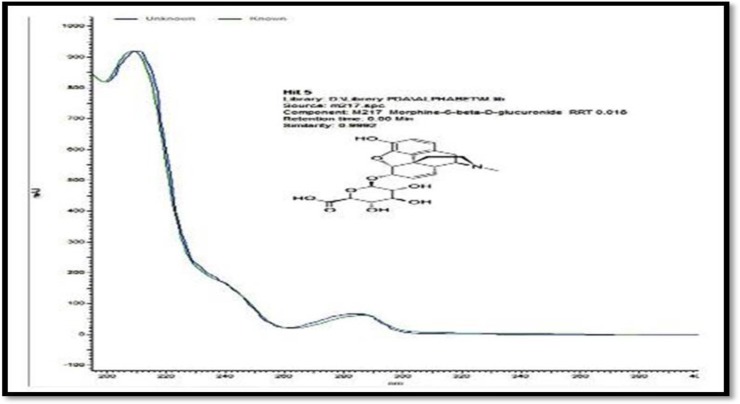
Ultraviolet Spectrum of morphine-6-glucuronid measured by HPLC it shows the morphine metabolism in the Rabbit’s liver

**Fig. 8.**
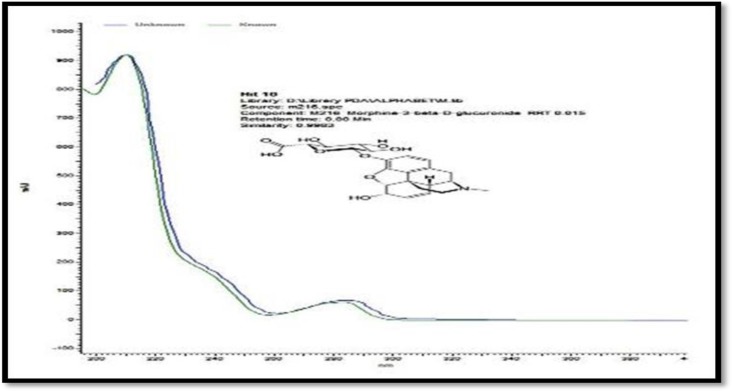
Ultraviolet Spectrum of morphine-3-glucuronide measured by HPLC it shows the morphine metabolism in the Rabbit’s liver

Quantitative analysis of carcass samples of treatment rabbit with concentrations of 25, 50, and 12.5mg/ml was indicative of concentration and metabolites difference in tissues for each concentration (Table 3, Figs. 6–8). Therefore, concentration gradient from the minimum to the maximum value: R3= 50mg/ml was found in liver, kidney, urine, muscle, bile, and spleen.

**Table 3.** Concentrations of morphine in Rabbit samples administered different dosages of morphine Sulfate via ear perfusion at 3h period, R0= control, R1= 12.5mg/ml, R2= 25mg/ml, R3= 50mg/ml

**Rabbit carcasses samples**	**R0**	**R1**	**R2**	**R3**

	**Mean Concentration (ng/g)**	**Mean Concentration (ng/g)**	**Mean Concentration (ng/g)**	**Mean Concentration (ng/g)**
**Liver**	0	47	215	2695
**Kidney**	0	28	505	2390
**Spleen**	0	0	722	1191
**Bile**	0	0	629.5	1411
**Mesenteric muscle**	0	31	500.7	1666
**Urine**	0	655.4	891	1969

At an R2= 25mg/ml the downtrend of this concentration slope was observed in the urine, spleen, bile, kidney, muscle, and liver respectively. In R1= 12.5mg/ml samples of urine, liver, muscle, kidney, spleen, and bile were measured and all specimens except spleen and bile were morphine.

Analysis of *C. albiceps* maggot samples settled on the treatment carcass with R3 morphine content was 210 and 146ng/g, R2 was 140 and 63ng/g respectively in peak feeding and post-feeding larvae. No morphine was recovered from post-feeding larvae fed at R1 concentrations. Adult *C. maxillosus* with concentrations of R1, R2 and R3 were lack of morphine in all treatment carcasses (Table 4).

**Table 4.** Concentration of morphine in *C. albiceps* larval and *C. maxillosus* administrated different dosage of morphine sulfate via ear perfusion at 3h period: R0= control, R1= 12.5mg/ml, R2= 25mg/ml, R3= 50mg/ml

**Insects**	**R0**	**R1**	**R2**	**R3**

	Mean Concentration (ng/g)	Mean Concentration (ng/g)	Mean Concentration (ng/g)	Mean Concentration (ng/g)
**Peak feeding larvae (*C.albiceps*)**	0	25.64	139.8	210
**Post feeding lar vae (*C.albiceps*)**	0	0	63.37	146
***C.maxillosus* (Adult)**	0	0	0	0

Statistical analysis confirmed this according to ANOVA test, since (P< 0.05) there is a significant difference between concentrations (Table 5). According Tukey HSD, we find homogeneous subsets and in the other words, different subsets are (R0, R1), (R1, R2), and R3.

**Table 5.** Tukey HSD analysis in rabbit carcasses administrated different dosages

**Tukey HSD^a^**	**Concentration**			

Rabbit samples	N	**Subset for alpha = 0.05**		

		1	2	3
R0	6	.0000		
R1	6	126.9000	126.9000	
R2	6		577.2000	
R3	6			1887.0000
P value		.914	.128	1.000

(R0= control, R1= 12.5mg/ml, R2= 25mg/ml, R3= 50mg/ml)

## Discussion

Morphine is a derivative of the opioid group. Opioids like morphine, heroin and tramadol have similar pharmacological properties. We have chosen morphine for this study because there are many of death in the world due to heroin or tramadol overdose. After absorption, morphine is rapidly distributed within the body. Morphine has a short half-life of approximately two hours. The main site of morphine metabolism is the liver. The process of elimination of morphine and heroin and codeine are similar in the body (23). Using a live animal model in toxicology analysis caused the results obtained to be closer to reality. We observed gradient concentration in various tissues of rabbit. We used *C. albiceps* (Diptera: Calliphoridae) and *C. maxillosus* (Coleoptera: Staphylinidae) as a toxicological bio-indicator. *Chrysomya albiseps* is first colonizer in tropical and sub-tropical areas particular in warm seasons (1, 25). The quantity of drug in tissues and *C. albiceps* larval stages seems to be dose-dependent.

The results of other studies also have attested to morphine concentration difference in various tissues (10, 13, 23, 26). It would be better to use the identical development stages of insect, especially peak feeding and post-feeding larvae in order to the toxicological analysis (26, 27). We also focused on the quantitative and qualitative analysis of the morphine on peak feeding and post feeding of *C. albiceps* species and adult *C. maxillosus*. Our findings showed that there was a difference in terms of concentration at various stages of larval development so that morphine concentration in peak feeding stage was higher than post-feeding larvae. Morphine content decreases during developmental process due to metabolism and drug elimination (4, 6, 11, 27, 28). Predator beetles are an appropriate matrix for the detecting of drugs and toxins (15). However, quantitative and qualitative analysis of the beetle (*C. maxillosus*) was not detectable. The amount of drug decreases with progress level of the food chain. In line with the results, other studies also take into account the changes in drug concentration caused by the difference in kinetics detoxification, drug stability, growth stage, insect species and the tissue of maggot placement (10, 12, 24, 29, 30).

Morphine is a polar compound and soluble in water to the (1/5000) and it is excreted through the nephritic system and Malpighian tubes. Therefore, morphine concentration in post feeding larval stage accompanied by stop feeding reduces against peak feeding. Other studies (10, 22, 24) confirming the results gained from this study showed that the rate of eliminating drug increases in post feeding larval stage.

In this study to extract of morphine from tissues, we used the acid digestion method. Acid digestion method for extracting the drug have preferred to enzyme digestion. The efficiency of acid digestion is 93% against 64% of enzymatic digestion (31). In studies conducted on the measurement of morphine on *L. sericata* larva with similar method, the results were slightly different compared to the current study (22, 23). These differences can stem from several factors such as extraction method, sensitivity of method, insect species and so forth. They used RIA device to analyze the morphine, while we applied HPLCUV. Besides our studied insect was *C. albiceps* but *L.sericata* was assessed in those studies. The volume of distribution in morphine is 3–5Kg/L. Molecules with volume of distribution (VD) >3 have redistribution after death and the concentration balance between blood and tissue changes after death (24).

According to our results, there was no linear relationship between morphine levels in carcass and larval stages feeding. This issue has been mentioned by some researchers (10, 23, 32). The necessity of review methods of validity was referred in toxicology (26). Drug detection occurs when the amount of absorption in the body exceeds the amount of drug elimination (4, 15). While this study showed that morphine will not bioaccumulate in the tissue.

Morphine concentration in treatment samples and larvae due to calculated amount for LOQ device illustrated that all obtained concentrations are statistically valuable. This study indicated necrophagous insects could be a valuable data in the investigation of death. Difference in the result of drug measurement in different methods of assay, only qualitative results such as presence of drug in insects can be valuable.

## Conclusion

Necrophagous insects on the carcass are a source of information from antemortem conditions. Moreover, using the entomotoxicological analysis can get information about the causes and manner of death. Therefore, the results of insect analysis are precisely the reflection of the carcass condition. Although there is no linear relationship between the concentration of morphine in the rabbit and insect tissue, however, using analysis of the necrophagous insect's tissue can be found to the presence of the drug (morphine) in the carcass.

## Footnotes

1 Retention factor
